# Dimensions of Anxiety, Age, and Gender: Assessing Dimensionality and Measurement Invariance of the State-Trait for Cognitive and Somatic Anxiety (STICSA) in an Italian Sample

**DOI:** 10.3389/fpsyg.2018.02345

**Published:** 2018-11-27

**Authors:** Leonardo Carlucci, Marley W. Watkins, Maria Rita Sergi, Fedele Cataldi, Aristide Saggino, Michela Balsamo

**Affiliations:** ^1^School of Medicine and Health Sciences, G. d’Annunzio University of Chieti–Pescara, Chieti, Italy; ^2^Department of Educational Psychology, Baylor University, Waco, TX, United States

**Keywords:** anxiety, depression, trait, state, invariance, multigroup confirmatory factor analysis

## Abstract

The State–Trait Inventory for Cognitive and Somatic Anxiety (STICSA) is a widely used measure of state and trait anxiety that permits a specific assessment of cognitive and somatic anxiety. Previous research provided inconsistent findings about its factor structure in non-clinical samples (e.g., hierarchical or bi-factor structure). To date, no psychometric validation of the Italian version of the STICSA has been conducted. Our study aimed to determine the psychometric functioning of the Italian version of the STICSA, including its dimensionality, gender and age measurement equivalence, and convergent/divergent validity in a large sample of community-dwelling participants (*N* = 2,938; 55.9% female). Through confirmatory factor analysis, the multidimensional structure of both State and Trait STICSA scales, with each including Cognitive and Somatic dimensions, was supported. Factor structure invariance was tested and established at configural, metric, and scalar levels for males and females. Additionally, full factorial measurement invariance was supported for the State scale across young, middle age, and old adult groups whereas the Trait scale was partially invariant across age groups. The STICSA also showed good convergent validity with concurrent anxiety measures (State-Trait Anxiety Inventory and Beck Anxiety Inventory), and satisfactory internal discriminant validity with two depression measures (Teate Depression Inventory and Beck Depression Inventory-II). Results provided support for the multidimensionality of the STICSA, as well as the generalizability of the State and Trait scales as independent measures of Cognitive and Somatic symptomatology across gender in the general population. Implications for research and personality and clinical assessment are discussed.

## Introduction

Anxietyis an emotional state defined by cognitive as well as somatic symptomatology such as feelings of tension, worried thoughts, increased blood pressure, sweating, derealization, and the anticipation of a future danger or threat (American Psychiatric Association, 2013). Anxiety symptoms are the most common of mental disorders and affect nearly 33% of adults at some point in their lives (Bandelow and Michaelis, 2015). In Italy, the prevalence in life of anxiety disorders is close to 11% (de Girolamo et al., 2006; Kessler et al., 2007).

The most widely documented results from psychiatric epidemiology are that anxiety symptoms develop from childhood and persist into adulthood if not detected and treated (Regier et al., 1990; Wittchen and Jacobi, 2005; Kessler et al., 2012; Bandelow and Michaelis, 2015), and that females are significantly more likely than males to develop anxiety disorders throughout the lifespan (ratio 1:2) (McLean and Anderson, 2009; McLean et al., 2011).

Historically, there has been considerable debate regarding the dimensionality of anxiety: it was considered unidimensional by Freud (1920) but characterized with both trait and state dimensions by contemporary researchers (Spielberger, 1985; Endler, 1997). The modern differentiation between trait and state anxiety has a long and controversial history (Allport and Odbert, 1936; Carr and Kingsbury, 1938; Zuckerman, 1960, 1983; Fridhandler, 1986; Endler and Kocovski, 2001; Heeren et al., 2018). A notable amount of research differentiates, for anxiety as well as other psychological states, between transitory emotion that varies in duration and is characterized by observable symptoms (i.e., state anxiety), and an individual’s unobservable disposition to experience elevated anxiety in response to threat (i.e., trait anxiety) (Spielberger, 1983, 1985; Endler and Kocovski, 2001; Pacheco-Unguetti et al., 2010; Heeren et al., 2018).

Trait anxiety has been extensively conceptualized as a fundamental dimension along which people differ (Allport and Odbert, 1936; Cattell, 1946; Eysenck, 1953). According to several personality trait taxonomies, trait anxiety has been variously theorized as negative emotionality (Tellegen, 1985), neuroticism (Costa and MacCrae, 1992; Ashton et al., 2004), low emotional stability (Goldberg, 1992), a risk factor for the development of anxious symptomatology (Weems et al., 2007), and comparable to anxiety sensitivity (Lilienfeld et al., 1993). On the other hand, state anxiety has been viewed as an emotional state that varies in duration depending of the presence of the provocative stimulus. According to this distinction, individuals high on trait anxiety are more likely to experience episodes of state anxiety (in terms of intensity, frequency, duration) than those low on trait anxiety (Heeren et al., 2018).

Nevertheless, the state-trait distinction has been labeled as arbitrary and based on weak assumptions, such as the minor difference in instructions included in anxiety measures divided into state and trait scales (e.g., “last week” versus “generally”) by several authors (Allen and Potkay, 1981; Zuckerman, 1983; Luthans et al., 2007). Therefore, state and trait anxiety could be considered as only interchangeable labels, representing two interconnected components. Ultimately, a trait index can be inferred from a state measurement (Allen and Potkay, 1981).

More recently, the distinction between cognitive and somatic anxiety symptoms has been explored (Steptoe and Kearsley, 1990; Ree et al., 2008; Waechter and Stolz, 2015). Clinical investigators have long considered the symptoms of anxiety to be phenomenologically heterogeneous and involving a wide array of physical, emotional, and cognitive components (Buss, 1962; Schalling et al., 1975; Steptoe and Kearsley, 1990). For example, anxiety was seen to involve somatic symptoms such as hyperventilation, sweating, and trembling as well as cognitive symptoms such as worry, intrusive thoughts, and lack of concentration (Ree et al., 2008). This cognitive/somatic distinction might better encompass all aspects included in the construct of anxiety and might allow treatment to be tailored for the predominant modality of anxiety experienced (e.g., cognitively orientated meditation versus self-instructional training with physiologically orientated relaxation) (Steptoe and Kearsley, 1990). Another controversial issue regarding anxiety is the overlap between anxiety and depression (Flint, 2005; Wetherell and Gatz, 2005; Bryant et al., 2008).

A considerable amount of research has emphasized that anxiety and depression share a common component of general distress in addition to components specific to each disorder (Clark and Watson, 1991; Watson et al., 1995a,b; Smoller and Tsuang, 1998; Costello et al., 2003; Hasler et al., 2004; Godfrey et al., 2005; Shafer, 2006; Ree et al., 2008). This finding was not surprising given the high comorbidity between anxiety and depression mood disorders (Watson et al., 1995a,b; Costello et al., 2003; Godfrey et al., 2005). In line with the tripartite model (Clark and Watson, 1991), aversive emotional states (fear, anger, guilt) are associated with both anxiety and depression; the lack of positive affect (feeling tired) is associated with depression whereas physiological hyperarousal (trembling, dizziness, shaking) with anxiety (Beck et al., 1988; Clark and Watson, 1991; Watson et al., 1995a,b).

### Assessment of Anxiety

Given the hypothetical multidimensional nature of anxiety and its manifold symptomatic manifestations, the assessment of anxiety represents a challenge for clinicians and researchers.

#### STAI

The most widely used self-rating measure for measuring anxiety in its trait and state components is Spielberger (1983) State-Trait Anxiety Inventory (STAI), but recent studies have raised doubts about the anxiety construct as measured by the STAI (Bieling et al., 1998; Caci et al., 2003; Bados et al., 2010; Balsamo et al., 2013c; Hill et al., 2013). According to these authors, the STAI can best be conceptualized as assessing negative affect, rather than a pure measure of anxiety. Indeed, the STAI has exhibited poor discriminatory power between anxiety and depression (Kabacoff et al., 1997; Bieling et al., 1998; Kennedy et al., 2001; Balsamo et al., 2013c; Bergua et al., 2016) and its scores have been more strongly correlated with a measure of depression than a measure of anxiety (Grös et al., 2007). In addition, its use appears to be particularly problematic among older adults, due to its length and format (McDonald and Spielberger, 1983; Dennis et al., 2007; Therrien and Hunsley, 2012; Balsamo et al., 2018).

#### STICSA

To overcome some of the issues associated with the use of the STAI, Ree et al. (2008) developed a new measure based on Spielberger’s (1966) conceptualization of state and trait anxiety, named the State–Trait Inventory for Cognitive and Somatic Anxiety (STICSA). The STICSA also contains subscales measuring somatic and cognitive symptom clusters. For example, the cognitive cluster aims to capture aspects of anxiety related to thoughts (e.g., difficulty concentrating, worry, intrusive thoughts), whereas the somatic cluster aims to capture features that directly relate to physical experiences (e.g., sweating, muscle tension, palpitations). Additionally, the use of balanced scales composed by separate groupings of cognitive and somatic anxiety items potentially facilitates the differentiation of anxiety from anxiety-like symptoms (i.e., symptoms caused by a medical condition). Therefore, the STICSA differs from most extant measures of anxiety which contain an overrepresentation of cognitive (like the State-Trait Anxiety Inventory) or somatic symptoms (like the Beck Anxiety Inventory), which makes it difficult to distinguish between anxiety, mood, and medical symptoms (Ree et al., 2008; Elwood et al., 2012; Deacy et al., 2016). The inclusion of both trait-state and somatic-cognitive clusters might allow the STICSA to better capture the heterogeneity of symptoms associated with anxiety disorders (Watson et al., 2005).

Previous research has demonstrated that the STICSA exhibited strong psychometric properties in both clinical and non-clinical samples of adults (Grös et al., 2007, 2010; Ree et al., 2008; Van Dam et al., 2013; Balsamo et al., 2015b; Roberts et al., 2016) and children (Deacy et al., 2016), as well as across African and European American samples (Lancaster et al., 2015).

Specifically, the STICSA has demonstrated sufficient to excellent values of internal consistency for both the State (α = 0.74–0.95) and Trait (α = 0.75–0.95) scales and test–retest reliability for the Trait scale (*r* = 0.60–0.66) among young and older adults, students, and clinical groups (Grös et al., 2007, 2010; Ree et al., 2008; Van Dam et al., 2013; Balsamo et al., 2015b; Deacy et al., 2016; Roberts et al., 2016). Concerning convergent and divergent validity, several studies revealed that STICSA, both at scale and dimension level, correlated at medium to high levels with other anxiety measures (i.e., Mood and Anxiety Symptom Questionnaire, Cognitive-Somatic Anxiety Questionnaire, avoidance measure, worry, and social anxiety) and at medium levels with depression self-report measures (i.e., Depression Anxiety Stress Scales, Beck Depression Inventory-II) (Grös et al., 2007; Ree et al., 2008). These studies highlighted the discriminant power of the STICSA, which allowed differentiation of anxiety from depression better than other anxiety measures, avoiding misdiagnosis, a fairly frequent problem in clinical practice (Therrien and Hunsley, 2012).

The STICSA was designed to tap two correlated subscales (State and Trait), each composed of two interrelated dimensions (Cognitive and Somatic). Accordingly, it produces four scores: State Cognitive, State Somatic, Trait Cognitive, and Trait Somatic. Additionally, all four scores might be combined to produce a total anxiety score, the two state scores could be combined to produce a state anxiety score, the two trait scores could be combined to produce a trait anxiety score, the two cognitive scores could be combined to produce a cognitive anxiety score, and the two somatic scores could be combined to produce a somatic anxiety score.

Given these possible scoring schemes, the structure of the STICSA can be conceptualized in several different ways. Not all of these conceptualizations have been considered in extant research. Ree et al. (2008) performed confirmatory factor analyses (CFA) on the trait and state scales separately among Australian students and adults, finding two correlated factors within each scale (i.e., cognitive and somatic). Grös et al. (2010) only examined the trait scale among American students and also found correlated cognitive and somatic factors. Grös et al. (2007) included both state and trait STICA items in their analysis of responses from Canadian psychiatric patients and U.S. college students. Given that trait and state items are identical (response instructions differentiate trait items as experienced “in general” and state items as experienced “right now”), Grös et al. (2007) allowed item error terms to correlate and found that four correlated factors [Somatic State (SS), Somatic Trait (ST), Cognitive State (CS), and Cognitive Trait (CT)] best fit their data. However, neither higher-order nor bifactor models were tested. Balsamo et al. (2015b) found a similar oblique four-factor structure among older Italian adults but did not allow correlated item errors and did not include higher-order or bifactor models. Roberts et al. (2016) also analyzed both trait and state STICSA items (among Canadian college students) and found support for a correlated four-factor model as well as a higher-order model with a global anxiety factor and four first-order factors. However, their models did not include correlated error terms across the state-trait items and they did not consider bifactor models. In contrast, Lancaster et al. (2015) did not find support for the oblique four-factor model among African American and European American university students. Unfortunately, Lancaster et al. (2015) failed to test other potential structural models.

Given the lack of clarity about the factor structure of the STICSA, the current study aimed to address evidence for the dimensionality of the STICSA on a large cross-age sample. Through a confirmatory methodology, we tested all the STICSA factor structure models found in the literature (hierarchical, bifactor, four-factor, and two factors models), to evaluate which model best represent the anxiety construct as conceptualized by this instrument.

Another unaddressed issue associated with the psychometric functioning of the STICSA is its measurement invariance across gender and age. Although studies of gender differences in anxiety have provided support for the higher prevalence rates of anxiety symptomatology and disorders among females across the life span, in both community and clinical samples (Lewinsohn et al., 1998; Egger et al., 2003; Bruce et al., 2005; McLean and Anderson, 2009; McLean et al., 2011), no studies have investigated the impact of gender differences on the measurement of anxiety with the STICSA. Additionally, studies on age differences in anxiety have provided support for quantitative and qualitative differences of presentation of anxiety symptomatology in younger and older adults (Blazer et al., 1991; Christensen et al., 1999; Balsamo et al., 2018), but no studies had investigated the impact of age differences on the measurement of anxiety by the STICSA. Accordingly, the second aim of this study was to provide evidence of measurement invariance of the STICSA across age and gender. Lastly, convergent and divergent validity of the STICSA was addressed to provide further evidence for the ability of STICSA scores to differentiate anxious from depressive symptoms.

## Materials and Methods

### Participants

Participants were 2,983 Italian adults, including 1,667 females (55.9%) and 1,316 males (44.1%), of whom 1,780 (59.7%) were undergraduate students. The sample’s mean age was 36.26 years (*SD* = 20.25 years). The mean age for men was 37.94 years (*SD* = 20.55 years), and 34.94 years (*SD* = 19.93 years) for women. The mean level of education was 11.87 (*SD* = 3.67) years. In order to address measurement invariance across age of the STICSA State and Trait scales, the sample was split into three age groups: 18–25 (*N*_total_ = 1,556; *N*_male_ = 624, *N*_female_ = 932), 26–50 (*N*_total_ = 675; *N*_male_ = 319, *N*_female_ = 356), and 51–99 (*N*_tota l_ = 743; *N*_male_ = 366, *N*_female_ = 377) years. A statistically significant association between Gender and Age groups was found [χ_(2)_ = 20.84, *p* < 0.001], suggesting how differences between groups potentially could be influenced by the proportion of males and females across the age groups, rather than chance.

### Procedure

The sample was recruited through advertisements (flyers, newspapers, and online ads) posted for established community groups (e.g., youth centers, church groups, university student associations) in Italian cities located in northern, central, and southern sections of the country. Part of the sample used here, took part in a study of anxiety, co-rumination, shame, young schema theory, personality, and eating disorders, described elsewhere (Saggino et al., 2017a; Picconi et al., 2018).

A battery of tests, randomly sequenced, was administered by a team of psychologists and researchers. Socio-demographic variables including age, gender, and education were also collected in the present study to provide a comprehensive framework of the participants’ characteristics. Given the high prevalence of individual differences in anxiety disorders, we considered gender and age variables in the following analyses (e.g., McLean et al., 2011). Participants who did not complete any of the STICSA items were excluded a-priori from all analyses. Inclusion criteria were: ages from 18 to 99 years and the ability to complete self-administered questionnaires. Exclusion criteria included marked cognitive impairment, a drug abuse disorder, diagnoses of psychotic disorders, and major disorders of the central nervous system (e.g., Alzheimer’s disease, Parkinson’s disease, epilepsy). For invariance analyses, pairwise deletion was used to deal with the missing data in the age or gender variables. For all other analyses, only complete questionnaire data were used.

Study participants contributed voluntarily and anonymously, and no honorarium was given for completing the assessments. Written informed consent was obtained from all participants before starting the administration, according to the Declaration of Helsinki. The ethics committee of the Department of Psychological Sciences, Health and Territory, University of Chieti, Italy, approved the study.

### Measures

#### State–Trait Inventory for Cognitive and Somatic Anxiety (STICSA)

The STICSA (Ree et al., 2008; for the Italian version see Balsamo et al., 2015b, 2016b) is a 21-item measure designed to assess cognitive (e.g., “I feel agonized over my problems,” “I think that others won’t approve of me”) and somatic (e.g., “My heart beats fast,” “My muscles are tense”) symptoms, both on Trait and State variations. In the Trait Anxiety subscale, the individual rates how often a statement is true in general (on a four-point Likert-type scale from 1 = *almost never at all* to 4 = *almost always*), whereas in the State Anxiety subscale, the examinee rates how she or he feels at the moment of assessment (on a four-point Likert-type scale from 1 = *not at all* to 4 = *very much*). In total, the overall scale is made up of four subscales: State–Somatic (SS), Trait–Somatic (TS), State–Cognitive (SC), and Trait–Cognitive (TC).

#### State-Trait Anxiety Inventory-Form Y (STAI-Y)

The STAI-Y (Spielberger and Gorsuch, 1983) is a self-report anxiety behavioral instrument composed of two separate 20-item subscales that measure trait (baseline) and state (situational) anxiety, resulting from a revision of the original Form X (Spielberger et al., 1970). The STAI trait subscale measures relatively stable individual differences in anxiety proneness; i.e., differences in the tendency to experience anxiety; and the STAI state subscale measures the transitory anxiety state; i.e., subjective feelings of apprehension, tension, and worry that vary in intensity and fluctuate based on the situation. Respondents are asked to rate each item on a 4-point Likert-type scale, ranging from 1 = *almost never* to 4 = *almost always*. The total score ranges from 20 to 80, with higher scores indicating greater anxiety. Internal consistencies of scores on the STAI-Y ranged from good to excellent in non-clinical and clinical samples (Stanley et al., 1996; Roberts et al., 2016; Balsamo et al., 2018). Adequate test-retest reliabilities (Stanley et al., 1996; Dennis et al., 2007), and construct validity have emerged in several studies in older adult outpatients with a variety of psychiatric disorders (Kabacoff et al., 1997; Dennis et al., 2007). In this study, coefficient alphas were 0.94 (95% CI 0.932–0.948) and 0.91 (95% CI 0.896–0.921), respectively for the State and Trait subscales.

#### Beck Anxiety Inventory (BAI)

The BAI (Beck et al., 1988) is a self-report inventory of 21 items with a focus on somatic symptoms of anxiety (i.e., nervousness, inability to relax) that was developed as a measure adept at discriminating between anxiety and depression. Respondents are asked to assess the degree of distress caused by these symptoms over the previous 7 days on a 4-point Likert-type scale, ranging from 0 = *not at all* to 3 = *severely*. The total score ranges from 0 to 63, with higher scores indicating greater anxiety. The BAI showed good internal and test–retest reliability as well as acceptable discriminative validity in samples of anxiety patients and non-clinical older adults (Beck et al., 1988; de Beurs et al., 1997; Diefenbach et al., 2009; Balsamo et al., 2018). Coefficient alpha for this study was 0.95 (95% CI 0.952–0.957).

#### Teate Depression Inventory (TDI)

The TDI is a 21-item self-report instrument designed to assess depressive symptoms (Balsamo and Saggino, 2013, 2014; Balsamo et al., 2014), as specified for major depressive disorder by the latest editions of the Diagnostic and Statistical Manual of Mental Disorders (DSM-IV-TR, DSM-5; American Psychiatric Association). It was developed via Rasch logistic analysis of responses in order to overcome inherent psychometric weaknesses of existing measures of depression (Balsamo and Saggino, 2007). Each item is rated on a five-point Likert-type scale, ranging from 0 = *always* to 4 = *never*. The TDI has exhibited strong psychometric properties in both clinical and non-clinical samples (Balsamo et al., 2013a,c, 2014, 2015a,c, 2016a; Innamorati et al., 2013; Saggino et al., 2017b, 2018; Carlucci et al., 2018; Contardi et al., 2018). In the present sample, Cronbach’s alpha was 0.91 (95% CI 0.907–0.917).

#### Beck Depression Inventory–II (BDI–II)

The BDI–II is a 21-item self-report inventory designed to assess the presence and severity of depressive symptoms, according to DSM-IV criteria (Beck et al., 1996). Each item is rated on a 4-point Likert-type scale ranging from 0 to 3, based on the severity of depressive symptoms over the last 2 weeks. Each item is a list of four statements arranged in increasing severity about a particular symptom of depression. The total score ranges from 0 to 63, with higher scores indicating more severe depressive symptoms. Several studies revealed high overall internal and test-retest reliability and validity for the BDI-II in undergraduates, psychiatric, and normal older adults (Gallagher et al., 1983; Beck et al., 1996; Dozois et al., 1998; Sprinkle et al., 2002; Titov et al., 2011). Coefficient alpha for this study was 0.83 (95% CI 0.817–0.873).

### Data Analysis

We conducted CFAs in our sample to test the eight structural models underlying items of the STICSA that have been employed in prior studies (see Models 1–8 in Supplementary Materials)^1^.

*Model 1* – *a one-factor model*, in which all items were forced to load on a single higher order factor (Grös et al., 2007);
*Model 2* – *a two factor oblique model* (CS-SS), in which items in the State scale loaded on either Cognitive and Somatic factors (Ree et al., 2008);
*Model 3* – *a two factor oblique model* (CT-ST), in which items in the Trait scale loaded on either Cognitive and Somatic factors (Ree et al., 2008);
*Model 4* – *a two factor oblique model* (S-T), in which items loaded on either State or Trait factors (Grös et al., 2007);
*Model 5* – *a two factor oblique model* (C-S), in which items loaded on Cognitive or Somatic factors (Grös et al., 2007);
*Model 6* – *a four factor oblique model*, in which the CT, ST, CS, SS subscales were directly modeled (Grös et al., 2007; Roberts et al., 2016);
*Model 7 – a bifactor model*, in which all STICSA items were forced to load both on a global anxiety factor and on 4 specific factors (CT, ST, CS, SS), corresponding to the STICSA subscales (Roberts et al., 2016);
*Model 8* – *a hierarchical model*, with one higher order factor and four first-order factors, the CT, ST, CS, SS.

The robust weighted least squares (WLSMV) method using a diagonal weight matrix and robust standard errors and a mean- and variance adjusted χ2 test statistic (Muthén, 1998; Muthén and Asparouhov, 2002; Muthén and Muthén, 2012b) was used to estimate parameters. The WLSMV is a robust estimator which does not assume normally distributed data (Brown, 2014) and seems to work well under a variety of conditions if sample size is 200 or better (Flora and Curran, 2004; Rhemtulla et al., 2012). Following Grös et al. (2007), in models 1 and 5, the error terms associated with corresponding items in the STICSA State and Trait were correlated. In these measurement models, the correlated error terms reflected a method effect (e.g., reversed/similarly worded items, acquiescence, or social desirability) (Marsh, 1996; Brown, 2014).

Model fit was assessed with the: (a) robust WLSMV chi-square (χ2) statistic and its degrees of freedom; (b) Tucker Lewis Index (TLI); (c) comparative fit index (CFI); and (d) root mean square error of approximation (RMSEA) and its 90% confidence interval (90% CI). Due to the large sample size, interpretation of the robust WLSMV chi-square square as a measure of fit was eschewed. An adequate fit between the target model and the observed data would produce TLI and CFI values of 0.90 and above, while values of 0.95 and above were considered to indicate excellent fit. RMSEA values of 0.08 or less were considered to reflect an adequate fit, while values of 0.05 or less were considered to reflect good fit (Schermelleh-Engel et al., 2003; Brown, 2014).

To examine factor structure invariance (*measurement invariance*) across gender and age, multigroup CFAs were performed according to Muthén and Muthén (2012b), using the WLSMV method and theta parameterization. Configural invariance is established when factor loadings and thresholds are free across groups, residual variances fixed at one in all groups, and factor means fixed at zero in all groups. In the metric invariance model, factor loadings are constrained to be equal across groups, residual variances fixed at one in one group and free in the other groups, and factor means fixed at zero in one group and free in the other groups. Scalar invariance models had factor loadings and thresholds constrained to be equal across groups, residual variances fixed at one in one group and free in the other groups, and factor means fixed at one in one group and free in the other groups. Given the large sample size, chi-square difference tests would be overly sensitive to even trivial differences (Little et al., 2007). Therefore, evaluation of invariance was based on the difference (Δ) of CFI and RMSEA indexes (Chen, 2007). A change of CFI ≥ -0.010 between consecutive models and a change of RMSEA ≥ 0.015 was considered as non-invariance (Chen, 2007). To investigate concurrent validity of test score interpretations, Pearson correlations were calculated between scores on the STICSA and scores on the STAI-Y, BAI (for the convergent validity), TDI, and BDI-II (for the discriminant validity). We also compared the STICSA and STAI pairs of correlation coefficients in the analysis of discriminant validity following Meng et al. (1992). This procedure involves performing a Fisher Z transformation on the correlation coefficients so that they can be compared via a *t*-test.

MPLUS v7 (Muthén and Muthén, 2012a) was used for the confirmatory factor analyses, SPSS V.22 (Corp, 2013) was used for all descriptives, correlations, and alpha reliability coefficients. Also, R Statistic for hierarchical McDonald omega (*h*ω) was used to estimate the reliability of the state and trait STICSA scales, since it is more accurate than coefficient alpha in multidimensional measures (Zinbarg et al., 2006; McDonald, 2013).

## Results

### Confirmatory Factor Analysis

As expected, due to the large sample size, the chi-squared index was found to be significant for all models. However, only models 2 and 3 exhibited acceptable fit to the data, suggesting that the State and Trait scale of the STICSA, with each including Cognitive and Somatic dimensions well represented our STICSA Italian adaptation structure (see Table 1). The degree of relationship (standardized λ weights) for each item with its correspondent first-order factors were all significant (*p* < 0.001) in these two models (see Supplementary Table 1).

**Table 1:** Fit indices for the structural models tested (*N* = 2,983).

Model	χ^2^	*df*	TLI	CFI	RMSEA	90% CI
Model 1: One factor with correlated error	17461.732^∗^	799	0.81	0.82	0.084	0.083–0.085
Model 2: Two state factors: SS and CS	2419.063^∗^	188	0.95	0.95	0.063	0.061–0.065
Model 3: Two trait factors: ST and CT	2589.648^∗^	188	0.94	0.94	0.065	0.063–0.068
Model 4: Two factors: State and trait	28848.444^∗^	818	0.69	0.71	0.107	0.106–0.108
Model 5: Two factors: Somatic and cognitive with correlated error	10112.356^∗^	797	0.89	0.90	0.063	0.062–0.064
Model 6: Four factors (ST, CT, SS, CS)	18244.376^∗^	813	0.81	0.82	0.085	0.084–0.085
Model 7: Bifactor with four group factors (ST, CT, SS, CS) and one general factor	25349.554^∗^	778	0.71	0.74	0.103	0.102–0.104
Model 8: Hierarchical structure	24872.698^∗^	815	0.74	0.75	0.099	0.098–0.101


*^∗^*p* < 0.001. *Model 7-8:* (Roberts et al., 2016)*; Model 2-3:* (Ree et al., 2008)*; Model 4-5-1:* (Grös et al., 2007).*

*ST, STICSA somatic trait; CT, STICSA cognitive trait; SS, STICSA somatic state; CS, STICSA cognitive state; df, degrees of freedom; TLI, tucker lewis index; CFI, comparative fit index; RMSEA, root-mean-square error of approximation; 90% CI, 90% confidence interval of RMSEA.*

In Model 2, the STICSA State scale item loadings on the SS-CS factors ranged from 0.55 to 0.88, with an average standardized factor loading of 0.73. Squared multiple correlations ranged from 0.30 to 0.78, with an average SMC of 0.54 indicating that, on average, 29% of the variance in observed variables was accounted for by latent factors. The latent factor correlations were high (0.73). In Model 3, the standardized factor loadings of the STICSA Trait items ranged from 0.49 to 0.79 for the CT-ST factors, with an average standardized factor loading of 0.67. Squared multiple correlations ranged from 0.25 to 0.64, with an average SMC of 0.46 indicating that, on average, 21% of the variance in observed variables was accounted for by latent factors. The latent factor correlations were high (75). In terms of local misfit, a careful inspection of the modification index did not suggested a respecification of either Model 2 or Model 3.

### Multigroup CFA

Tests of measurement invariance across gender and age were examined through a multiple-group confirmatory factor analysis. Based on the previous findings, Models 2 and 3 were used as baseline models and tested for the data fit across: (a) male versus female groups for the first comparison and (b) age groups (18–25; 26–50; 51–99 years) for the second comparison. Following the (Muthén and Muthén, 2012b, p. 545) sequential procedures in each comparison, the fit of Models 2 and 3 were first tested separately in groups. Then, restrictive models were used to test for equal form (configural invariance), equal factor loadings (metric invariance), and equal indicator thresholds and residual variances (scalar invariance). Results of these measurement invariance analyses are presented in Table 2.

**Table 2:** Tests of measurement invariance across gender and age.

Model	χ^2^	*df*	Δ*df*	TLI	CFI	ΔCFI	RMSEA	90% CI	ΔRMSEA
**GENDER^†^**									
**STATE**									
**Single-group solutions**									
*Male* (*n* = 1,316)	1011.724^∗^	188		0.957	0.962		0.058	0.054–0.061	
*Female* (*n* = 1,667)	1468.362^∗^	188		0.941	0.947		0.064	0.061–0.067	
Configural	2497.532^∗^	376		0.948	0.953		0.062	0.059–0.064	
Metric	2560.230^∗^	394	18	0.949	0.953	0.000	0.061	0.058–0.063	-0.001
Scalar	2380.610^∗^	439	45	0.959	0.957	-0.004	0.054	0.052–0.057	-0.007
TRAIT									
Single-group solutions									
*Male* (*n* = 1,316)	1133.253^∗^	188		0.943	0.949		0.062	0.058–0.065	
*Female* (*n* = 1,667)	1573.278^∗^	188		0.927	0.934		0.067	0.064–0.070	
Configural	2719.534^∗^	376		0.933	0.940		0.065	0.062–0.067	
Metric	2841.328^∗^	394	18	0.934	0.938	-0.002	0.065	0.062–0.067	0.000
Scalar	2789.981^∗^	439	45	0.943	0.940	0.002	0.060	0.058–0.062	0.005
**AGE^‡^**									
**STATE**									
**Single-group solutions**									
*Age 18–25* (*n* = 1,556)	1306.368^∗^	188		0.949	0.954		0.062	0.059–0.065	
*Age 26–50* (*n* = 675)	600.368^∗^	188		0.956	0.961		0.057	0.052–0.062	
*Age 51–99* (*n* = 742)	713.986^∗^	188		0.951	0.956		0.061	0.057–0.066	
Configural	2606.486^∗^	564		0.949	0.954		0.060	0.058–0.063	
Metric	2904.808^∗^	664	100	0.952	0.950	0.004	0.058	0.056–0.061	0.002
Scalar	2674.628^∗^	711	47	0.961	0.956	-0.006	0.053	0.051–0.055	-0.005
**TRAIT**									
**Single-group solutions**									
*Age 18–25* (*n* = 1,556)	1557.729^∗^	188		0.924	0.932		0.068	0.065–0.072	
*Age 26–50* (*n* = 675)	589.854^∗^	188		0.949	0.954		0.056	0.051–0.061	
*Age 51–99* (*n* = 742)	604.609^∗^	188		0.958	0.962		0.055	0.050–0.060	
Configural	2745.246^∗^	564		0.937	0.944		0.062	0.060–0.065	
Metric	3377.579^∗^	664	100	0.934	0.930	0.014	0.064	0.062–0.066	0.002
Scalar	4015.815^∗^	711	47	0.925	0.915	0.015	0.068	0.066–0.071	0.004


**df*, degrees of freedom; TLI, tucker lewis index; CFI, comparative fit index; RMSEA, root-mean-square error of approximation; 90% CI = 90% confidence interval of RMSEA.*

*^∗^*p* < 0.001.*

*^†^*N* = 2,983.*

*^‡^*N* = 2,974, from the total sample we excluded nine cases with missing values in age variable.*

Configural, metric, and scalar invariance was demonstrated across male and female groups. As seen in Table 2, the ΔCFI were lower than |0.010| and RMSEA were lower than |0.015| for all the comparisons, therefore the assumption of equal factor loadings and indicator thresholds in males and females group were confirmed for Models 2 and 3. However, the χ^2^difference between all models tested was found to be significant (*p* < 0.001), both at State (Model 2) and at Trait (Model 3) scale of STICSA.

For age groups, measurement invariance was found for Models 2 and 3 in each of the three groups, separately. Subsequently, the adequacy of the same models was examined through the amount of configural, metric, and scalar invariance simultaneously in the three age groups (18–25; 26–50; 51–99 years old). Fit indices in general supported an adequate model fit for configural, metric, and scalar invariance across age for Model 2 (State scale of STICSA, with Cognitive and Somatic subscales). Configural invariance was also established for Model 3 across the three age groups. Fit indices showed that significant differences across the age groups were found on factor loadings, item thresholds, and residual levels for Model 3. All ΔCFI were greater than |0.01| cut-off criteria; therefore, metric and scalar invariance between the age groups was not confirmed for model 3. A careful inspection of modification index (MI) revealed that the factor loadings of items 10–11 and 20, respectively, for the first (18–25 years) and third age group (51–99 years), differed significantly between groups.

### Descriptives and Concurrent Validity

Means, standard deviations, correlations, and internal consistency are reported in Table 3. The reliability estimates for the STICSA subscale were high, with ω coefficients of 0.96 and 0.94, respectively, for State and Trait total scores. In order to investigate the concurrent and discriminant validity of the STICSA, one-tailed correlations among the STICSA dimensions with other measure of anxiety and measures of depression were computed (Table 3).

**Table 3:** Descriptives, correlations and reliabilities.

	Mean	SD	α (ω)	1	2	3	4	5	6	7	8	9
1. STICSA-Trait, somatic	18.62	5.69	(0.89)		0.644^∗∗^	0.715^∗∗^	0.554^∗∗^	0.451^∗∗^	0.502^∗∗^	0.571^∗∗^	0.422^∗∗^	0.354^∗∗^
2. STICSA-Trait, cognitive	19.10	6.08	(0.90)			0.465^∗∗^	0.780^∗∗^	0.417^∗∗^	0.598^∗∗^	0.735^∗∗^	0.577^∗∗^	0.603^∗∗^
3. STICSA-State, somatic	16.21	5.61	(0.92)				0.626^∗∗^	0.421^∗∗^	0.542^∗∗^	0.483^∗∗^	0.354^∗∗^	0.248^∗∗^
4. STICSA-State, cognitive	17.17	6.28	(0.91)					0.386^∗∗^	0.699^∗∗^	0.634^∗∗^	0.538^∗∗^	0.592^∗∗^
5. BAI^†^	12.65	12.64	0.95						0.489^∗∗^	0.583^∗∗^	0.442^∗∗^	0.387^∗∗^
6. STAI-Y State^‡^	37.38	11.51	0.94							0.751^∗∗^	0.548^∗∗^	.b
7. STAI-Y Trait^‡^	39.95	10.73	0.91								0.706^∗∗^	.b
8. TDI	27.95	13.09	0.91									0.670^∗∗^
9. BDI-II^§^	11.29	8.57	0.83									


*^†^N = 722.*

*^‡^N = 444.*

*^§^ N = 278.*

**.b* Cannot be computed because there are not enough subjects who completed the BDI-II scale.*

*^∗∗^*p* < 0.001 (one-tailed).*

*STICSA, state-trait inventory for cognitive and somatic anxiety; BAI, beck anxiety inventory; STAI-Y, state-trait anxiety inventory – form Y; TDI, teate depression inventory; BDI-II, beck depression inventory-II.*

#### Convergent Validity

As expected, all the STICSA scales were highly inter-correlated (ranged from *r* = 0.916 to *r* = 0.465, *p* < 0.001, respectively, for STICSA Trait Somatic and STICSA Trait Cognitive). Additionally, STICSA Trait and State scale dimensions were medium to highly correlated with the STAI-Y scales (from *r* = 0.502 to *r* = 0.699, *p* < 0.001, and from *r* = 0.483 to *r* = 0.735, *p* < 0.001, respectively, for STAI-State and STAI-Trait), but moderately correlated with BAI total scores (from *r* = 0.386, to *r* = 0.417, *p* < 0.001, respectively, for STICSA State Cognitive and STICSA Trait Cognitive).

#### Discriminant Validity

All anxiety dimensions used in this study correlated moderately with depression measures. Notably, the Somatic subscale of the Trait STICSA was correlated with depression measures (*r*_TDI_ = 0.422, *r*_BDI-II_ = 0.354; *p* < 0.001); as well as the Somatic subscale of the State scale (*r*_TDI_ = 0.354, *r*_BDI-II_ = 0.248; *p* < 0.001). No correlations were computed between the STAI-Y and the BDI-II, given the low number of participants who completed the BDI-II.

Subsequently, correlation coefficients between the STICSA State and Trait dimensions and STAI Trait and State anxiety and the TDI depression were statistically compared (Meng et al., 1992). Comparisons revealed that the STAI Trait correlated more highly with TDI scores than with STICSA Trait somatic, and cognitive subscales [*t*_(441)_ = 9.09, *p* < 0.01, *Z* = 8.38; *t*_(441)_ = 5.29, *p* < 0.01, *Z* = 5.12, respectively]. Similarly, the STAI State scale correlated more highly with TDI scores than with STICSA-State somatic subscale scores [*t*_(441)_ = 5.10, *p* < 0.01, *Z* = 4.93]. No differences were found between the STAI State and STICSA State cognitive with the TDI [*t*_(441)_ = 0.33, *p* = 0.37, *Z* = 0.33, respectively]. These results, in line with previous research (Grös et al., 2007), indicated that the STICSA State somatic, Trait scale, and cognitive and somatic subscales were better measures of anxiety than depression.

## Discussion

The STICSA was developed to overcome the psychometric weakness of existing instruments of anxiety based on the distinction between trait and state anxiety (i.e., the STAI), such as their extensive overlap with depression (Caci et al., 2003; Balsamo et al., 2013c; Roberts et al., 2016). Even though the STICSA State and Trait scale and subscales have exhibited high internal consistency reliability, as well as construct consistent correlations in patients, controls, and community groups (Grös et al., 2007; Ree et al., 2008; Van Dam et al., 2013), no consensus was found in the literature about the factor structure of the STICSA (Ree et al., 2008; Lancaster et al., 2015; Roberts et al., 2016).

The present study, firstly, provided further evidence that scores from the Italian adaptation of the STICSA were reliable and valid measures of multidimensional (cognitive and somatic) anxiety in a non-clinical population. Consistent with some previous research, the confirmatory factor analysis confirmed the STICSA factor structure of the Trait and State scales as separate measures of anxiety (Ree et al., 2000, 2008; Deacy et al., 2016). Each of the State and Trait forms was composed of two latent and correlated factors, thereby lending support to the distinction between cognitive and somatic aspects of anxiety. No support was found for the hierarchical and bifactor model of the STICSA scores with a global anxiety factor and four specific factors corresponding to the four subscales of the STICSA (trait/state; cognitive/somatic) (Roberts et al., 2016), nor for an oblique four-factor model of STICSA scores with factors corresponding to the somatic and cognitive subscales of the state and trait versions of the STICSA previously found in an elderly population (Balsamo et al., 2015b). This result was not in accordance with the increasing number of studies which have supported a bifactor structure for psychopathological scales (Al-Turkait and Ohaeri, 2010; Kriston et al., 2012; Saggino et al., 2018; Wang et al., 2018).

The second aim of the study was to assess the measurement equivalence of the STICSA scores across males and females, and across young, middle age, and older adult samples in order to determine whether scores between these groups could be interpreted with confidence. For gender comparisons, results indicated that the STICSA State and Trait scale items showed the same consistency in factor structure across male and female respondents. Given the empirical evidence that females have demonstrated greater negative affectivity (such as trait anxiety) and higher rates of anxiety disorders and symptomatology (Kessler et al., 1994; Breslau et al., 2000) than men across the life span (Lewinsohn et al., 1998; McLean et al., 2011), this finding appears to be interesting since the STICSA factor structure it was found invariant across gender in this study. Full measurement invariance across gender suggested that the proposed factor structure, pattern of factor loadings, and thresholds of STICSA State and Trait scales were similar for males and female respondents in this study. Therefore, STICSA State and Trait scores appear to reflect true gender differences in anxiety constructs and can be used interchangeably in males and females (Brown, 2014).

Concerning age, only the STICSA State scale was found to be invariant at the configural, metric, and scalar levels. Metric and scalar measurement equivalence was not found across age groups for the STICSA Trait scale. There is a general consensus in the literature on the impact of age in developing anxious symptomatology (Christensen et al., 1999; Balsamo et al., 2018). The nature of the anxiety experienced by older individuals may differ qualitatively from younger ones. For instance, older people reported greater ability to control their emotions (Lawton et al., 1993) and greater level of worry about health; whereas younger adults experienced worry about finances and family and tended to report more negative affect.

In our sample, younger, middle-aged, and older adult groups interpreted and responded to the Trait scale of the STICSA with significant variability between them. They differed significantly about how much of the latent trait was required to endorse an item. Great age-variability was found across items that assess somatic conditions (“*Butterflies in the stomach*”), and cognitive process (“*Can’t get thoughts out of mind*” and “*Trouble remembering things*”). Given these age differences, clinicians might misrepresent cognitive and somatic symptomatology or over/underestimate the magnitude of state anxiety reactions under stressful circumstances across gender groups (Ree et al., 2008). Therefore, future research should examine in detail the capacity of these items in discriminating trait anxiety across age.

In line with previous research, all the STICSA Trait and State scores of the Cognitive and Somatic scales were highly inter-correlated (Ree et al., 2000, 2008; Grös et al., 2007; Balsamo et al., 2015b; Roberts et al., 2016). The STICSA showed good convergent validity with the STAI, moderate convergent validity with the BAI, and satisfactory discriminant validity with the TDI and the BDI-II. In line with the Clark and Watson (1991) tripartite model, our results suggested that anxiety and depression shared a non-specific component of generalized distress (negative affect). In addition, STICSA State and Trait measures of Cognitive and Somatic symptoms were more specific to anxiety (i.e., physiological hyperarousal) than depression compared to the STAI. Similarly, the moderate to strong correlations between STICSA (and its subscale) scores and concurrent measures of anxiety in this study provided further evidence of STICSA scales as a pure measure of anxiety (Innamorati et al., 2013).

Limitations of the present study included the characteristics of our sample and the specific measures selected for the validity analyses. The use of a convenience sample, composed of non-clinical participants (mostly undergraduate students), potentially limits the generalizability of this study (Peterson and Merunka, 2014). Additionally, the inclusion of student data in research might have introduced uncontrolled systematic variance components (Balsamo, 2010, 2013; Balsamo et al., 2013b; Innamorati et al., 2014). As the specific measures selected for the validity analyses the STICSA was investigated exclusively in relation to a measure of general anxiety (i.e., BAI), neglecting measures of specific anxiety disorder (i.e., Panic Attack and Anticipatory Anxiety Scale; the Anxiety Sensitivity Index). Another limitation was reliance on unbalance samples to perform the correlation analyses between the measures. This is, partially, due to the sample recruitment strategy. Further studies could address the issue of comorbidity in clinical samples, controlling the STICSA State and Trait scale scores for depression (i.e., MIMIC models) or including depression as a covariate in regression models.

Regardless of these limitations, the current study demonstrated that STICSA scores are psychometrically reliable and valid measures that discriminated anxiety from depression in a non-clinical Italian population. The ability of STICSA to distinguish State and Trait dimensions of Cognitive and Somatic anxiety could provide a helpful opportunity for clinicians to: (a) perform an accurate differential diagnosis (e.g., discriminating anxiety from somatic symptomatology in oncology and geriatrics as well as in medical conditions); (b) promote recognition and effective treatment of anxiety disorders and comorbid disorders; (c) prove the efficacy of certain treatments in reducing specific anxiety symptoms. Future research should examine this discriminant power in association with specific symptoms of anxiety.

## Author Contributions

LC and MB designed the study and conducted the statistical analyses. LC, MW, AS, and MB interpreted the data. LC, MB, and MW drafted the manuscript. MS and FC recruited the sample and collaborated in editing the final manuscript. All authors contributed toward data analysis, drafting and revising the paper, and agreed to be accountable for all aspects of the work.

## Conflict of Interest Statement

The authors declare that the research was conducted in the absence of any commercial or financial relationships that could be construed as a potential conflict of interest. The MR declared a past co-authorship with several of the authors LC, MS, AS, MB to the handling Editor.

## Footnotes

1 A series of additional models were also tested (i.e., Orthogonal two factor: State-Trait model; higher-order and bi-factor versions of the two-factor models). These additional models were under-identified or did not reached convergence. Therefore, they were considered unreliable and not informative, so they were not included in the present study.
