# Supplementary material for: Dimensions of Anxiety, Age, and Gender: Assessing Dimensionality and Measurement Invariance of the State-Trait for Cognitive and Somatic Anxiety (STICSA) in an Italian Sample
Source: Front Psychol. 2018 Nov 27;9:2345. doi: 10.3389/fpsyg.2018.02345 (PMC6277473; doi:10.3389/fpsyg.2018.02345)
Supplement: Supplementary file 1 [file Table_1.docx]

| Supplementary Table 1. Standardized and Unstandadized Factor Loadings and Standard Errors for the Models 2 and 3. | | | | | | |
| --- | --- | --- | --- | --- | --- | --- |
| STICSA Item |  | State | |  | Trait | |
|  |  | *State–Somatic* | *State–Cognitive* |  | *Trait–Somatic* | *Trait–Cognitive* |
| 1. Heart beats fast. |  | .627 (.015) [1.00] |  |  | .562 (.015) [1.00] |  |
| 2. Muscles are tense. |  | .733 (.012) [1.17] |  |  | .651 (.013) [1.16] |  |
| 3. Feel agonized over problems. |  |  | .778 (.010) [1.00] |  |  | .723 (.011) [1.00] |
| 4. Think others won’t approve. |  |  | .715(.013) [0.92] |  |  | .653 (.014) [0.90] |
| 5. Can’t make up mind. |  |  | .691(.012) [0.89] |  |  | .637 (.013) [0.88] |
| 6. Feel dizzy. |  | .755 (.013) [1.21] |  |  | .703 (.013) [1.25] |  |
| 7. Muscles feel weak. |  | .786 (.011) [1.25] |  |  | .717 (.012) [1.27] |  |
| 8. Feel trembly and shaky. |  | .883 (.010) [1.41] |  |  | .797 (.012) [1.42] |  |
| 9. Picture future misfortunes. |  |  | .784 (.011) [1.01] |  |  | .736 (.011) [1.02] |
| 10. Can’t get thoughts out of mind. |  |  | .713 (.012) [0.92] |  |  | .676 (.012) [0.93] |
| 11. Trouble remembering things. |  |  | .604 (.016) [0.78] |  |  | .519 (.016) [0.72] |
| 12. Face feels hot. |  | .689 (.015) [1.09] |  |  | .650 (.014) [1.16] |  |
| 13. Think worst will happen. |  |  | .837 (.011) [1.08] |  |  | .778 (.011) [1.08] |
| 14. Arms and legs feel stiff. |  | .817 (.011) [1.30] |  |  | .769 (.012) [1.37] |  |
| 15. Throat feels dry. |  | .671 (.015) [1.07] |  |  | .676 (.013) [1.20] |  |
| 16. Avoid uncomfortable thoughts. |  |  | .706 (.012) [0.91] |  |  | .625 (.013) [0.87] |
| 17. Irrelevant thoughts intruding. |  |  | .809 (.009) [1.04] |  |  | .763 (.010) [1.06] |
| 18. Breathing is fast and shallow. |  | .769 (.014) [1.23] |  |  | .736 (.013) [1.31] |  |
| 19. Cannot control thoughts. |  |  | .803 (.010) [1.03] |  |  | .775 (.010) [1.07] |
| 20. Butterflies in the stomach. |  | .572 (.020) [0.91] |  |  | .498 (.018) [0.88] |  |
| 21. Palms feel clammy. |  | .555 (.018) [0.89] |  |  | .510 (.018) [0.91] |  |
| *Note.* STICSA = State–Trait Inventory for Cognitive and Somatic Anxiety, () = standard errors, and [] = unstandardized factor loadings.  All factor loadings were significant at *p* < .001. | | | | | | |
