# Supplementary material for: Dimensions of Anxiety, Age, and Gender: Assessing Dimensionality and Measurement Invariance of the State-Trait for Cognitive and Somatic Anxiety (STICSA) in an Italian Sample
Source: Front Psychol. 2018 Nov 27;9:2345. doi: 10.3389/fpsyg.2018.02345 (PMC6277473; doi:10.3389/fpsyg.2018.02345)
Supplement: Supplementary file 2 [file Data_Sheet_1.docx]

**Model 1: One factor with correlated error**

STIC-T-3

STIC-S-3

STIC-T-4

STIC-T-5

STIC-T-9

STIC-T-10

STIC-T-11

STIC-T-13

STIC-T-16

STIC-T-17

STIC-T-19

STIC-T-1

STIC-T-2

STIC-T-6

STIC-T-7

STIC-T-8

STIC-S-4

STIC-S-5

STIC-S-9

STIC-S-10

STIC-S-11

STIC-S-13

STIC-S-16

STIC-S-17

STIC-S-19

STIC-S-1

STIC-S-2

STIC-S-6

STIC-S-7

STIC-T-12

STIC-S-8

STIC-S-12

STIC-T-14

STIC-S-14

STIC-T-15

STIC-S-15

STIC-T-18

STIC-S-18

STIC-T-20

STIC-S-20

STIC-T-21

STIC-S-21

**Model 2. Two-Factor State Model** **Model 3. Two-Factor Trait Model**

STIC-S-3

STIC-S-4

STIC-S-5

STIC-S-9

STIC-S-10

STIC-S-11

STIC-S-13

STIC-S-16

STIC-S-19

STIC-S-17

STIC-S-1

STIC-S-2

STIC-S-6

STIC-S-7

STIC-S-8

STIC-S-12

STIC-S-14

STIC-S-18

STIC-S-15

STIC-S-20

STIC-S-21

STIC-T-3

STIC-T-4

STIC-T-5

STIC-T-9

STIC-T-10

STIC-T-11

STIC-T-13

STIC-T-16

STIC-T-17

STIC-T-19

STIC-T-1

STIC-T-2

STIC-T-6

STIC-T-7

STIC-T-8

STIC-T-12

STIC-T-14

STIC-T-15

STIC-T-18

STIC-T-20

STIC-T-21

**Model 4. Two-Factor Trait-State Model**

STIC-T-3

STIC-T-4

STIC-T-5

STIC-T-9

STIC-T-10

STIC-T-11

STIC-T-13

STIC-T-16

STIC-T-19

STIC-T-17

STIC-T-1

STIC-T-2

STIC-T-7

STIC-T-6

STIC-T-8

STIC-T-12

STIC-T-15

STIC-T-14

STIC-T-18

STIC-T-20

STIC-T-21

STIC-S-3

STIC-S-4

STIC-S-5

STIC-S-9

STIC-S-10

STIC-S-11

STIC-S-13

STIC-S-16

STIC-S-19

STIC-S-17

STIC-S-1

STIC-S-2

STIC-S-7

STIC-S-6

STIC-S-8

STIC-S-12

STIC-S-15

STIC-S-14

STIC-S-18

STIC-S-20

STIC-S-21

**Model 5. Two-Factor Somatic-Cognitive Model**

STIC-T-1

STIC-T-2

STIC-T-6

STIC-T-7

STIC-T-8

STIC-S-1

STIC-S-2

STIC-S-6

STIC-S-7

STIC-T-12

STIC-S-8

STIC-T-14

STIC-S-14

STIC-T-15

STIC-S-15

STIC-T-18

STIC-S-18

STIC-T-20

STIC-S-20

STIC-T-21

STIC-S-21

STIC-S-12

STIC-T-3

STIC-S-3

STIC-T-4

STIC-T-5

STIC-T-9

STIC-T-10

STIC-T-11

STIC-T-13

STIC-T-16

STIC-T-17

STIC-T-19

STIC-S-4

STIC-S-5

STIC-S-9

STIC-S-10

STIC-S-11

STIC-S-13

STIC-S-16

STIC-S-17

STIC-S-19

**Model 6. Four-Factor Model**

STIC-T-3

STIC-T-10

STIC-T-4

STIC-T-5

STIC-T-9

STIC-T-11

STIC-T-13

STIC-T-16

STIC-T-17

STIC-T-19

STIC-T-1

STIC-T-2

STIC-T-6

STIC-T-7

STIC-T-8

STIC-T-12

STIC-T-14

STIC-T-18

STIC-T-15

STIC-T-20

STIC-T-21

STIC-S-3

STIC-S-4

STIC-S-5

STIC-S-9

STIC-S-10

STIC-S-11

STIC-S-13

STIC-S-16

STIC-S-19

STIC-S-17

STIC-S-1

STIC-S-2

STIC-S-6

STIC-S-7

STIC-S-8

STIC-S-12

STIC-S-14

STIC-S-18

STIC-S-15

STIC-S-20

STIC-S-21

**Model 7. Bifactor Model**

STIC-T-3

STIC-T-4

STIC-T-5

STIC-T-9

STIC-T-10

STIC-T-11

STIC-T-13

STIC-T-16

STIC-T-19

STIC-T-17

STIC-T-7

STIC-T-1

STIC-T-2

STIC-T-6

STIC-T-8

STIC-T-12

STIC-T-15

STIC-T-14

STIC-T-18

STIC-T-20

STIC-T-21

STIC-S-3

STIC-S-4

STIC-S-5

STIC-S-9

STIC-S-10

STIC-S-11

STIC-S-13

STIC-S-16

STIC-S-17

STIC-S-19

STIC-S-1

STIC-S-2

STIC-S-7

STIC-S-6

STIC-S-8

STIC-S-12

STIC-S-15

STIC-S-14

STIC-S-18

STIC-S-20

STIC-S-21

**Model 8. Hierarchical STICSA Model**

STIC-T-3

STIC-T-4

STIC-T-5

STIC-T-9

STIC-T-10

STIC-T-11

STIC-T-13

STIC-T-16

STIC-T-19

STIC-T-17

STIC-T-7

STIC-T-1

STIC-T-2

STIC-T-6

STIC-T-8

STIC-T-12

STIC-T-15

STIC-T-14

STIC-T-20

STIC-T-18

STIC-T-21

STIC-S-3

STIC-S-4

STIC-S-5

STIC-S-9

STIC-S-10

STIC-S-11

STIC-S-13

STIC-S-16

STIC-S-17

STIC-S-19

STIC-S-1

STIC-S-2

STIC-S-7

STIC-S-6

STIC-S-8

STIC-S-12

STIC-S-15

STIC-S-14

STIC-S-20

STIC-S-18

STIC-S-21
